# Retraction Note: Suppression of fumarate hydratase activity increases the efficacy of cisplatin-mediated chemotherapy in gastric cancer

**DOI:** 10.1038/s41419-023-05604-7

**Published:** 2023-01-20

**Authors:** Hong-En Yu, Feng Wang, Fang Yu, Zhao-Lei Zeng, Yun Wang, Yun-Xin Lu, Ying Jin, De-Shen Wang, Miao-Zhen Qiu, Heng-Ying Pu, Tie-Bang Kang, Dan Xie, Huai-Qiang Ju, Rui-Hua Xu, Hui-Yan Luo

**Affiliations:** 1 State Key Laboratory of Oncology in South China, Collaborative Innovation Center for Cancer Medicine, Sun Yat-sen University Cancer Center, Guangzhou, 510060 P. R. China; 2 Department of Medical Oncology, Sun Yat-sen University Cancer Center, Guangzhou, P. R. China; 3 Department of Health Examination, The Third Affiliated Hospital of Sun Yat-sen University, Guangzhou, 510700 P. R. China

Retraction to: *Cell Death and Disease* 10.1038/s41419-019-1652-8, published online 28 May 2019

The Editors-in-Chief have retracted this article due to an overlap in the western blot images in figures 4G and 6C. The Editors-in-Chief have therefore lost confidence in the reliability of the results of this study. None of the authors agree to this retraction.

